# A Potential Association Between Retinal Changes, Subjective Memory Impairment, and Anxiety in Older Adults at Risk for Alzheimer’s Disease: A 27-Month Pilot Study

**DOI:** 10.3389/fnagi.2019.00288

**Published:** 2019-10-29

**Authors:** Derrick L. Cheng, Louisa Thompson, Peter J. Snyder

**Affiliations:** ^1^The Warren Alpert Medical School of Brown University, Providence, RI, United States; ^2^Department of Neurology, Alpert Medical School, Brown University, Providence, RI, United States; ^3^The Interdisciplinary Neuroscience Program, University of Rhode Island, Kingston, RI, United States

**Keywords:** preclinical Alzheimer’s disease, subjective memory impairment, retinal nerve fiber layer, retinal biomarkers, Alzheimer’s disease, subjective cognitive decline

## Abstract

**Introduction:**

The utility of subjective memory impairment (SMI) as a risk marker for preclinical Alzheimer’s disease (AD) remains unclear; however, recent studies have identified a correlation between retinal biomarkers and onset of preclinical disease. This study examines the relationship between retinal biomarkers that have been associated with cerebral amyloid, an early hallmark of AD, and SMI scores in patients at risk for developing AD.

**Methods:**

Forty-nine cognitively normal subjects were followed over 27 months and evaluated using a combination of neuropsychological, psychological, and retinal imaging instruments. Subjective memory testing was conducted using the memory assessment clinic questionnaire (MACQ) and Depression, Anxiety, and Stress Scales (DASS). Multivariate linear analysis was conducted using STATA software.

**Results:**

Positive correlations were found between retinal nerve fiber layer (RNFL) volume and scores obtained from the MAC-Q at 27 months (MAC-Q_27), the DASS questionnaire for anxiety at 27 months (DASS-A_27), and the change in DASS-A over 27 months (dDASSA). There was also a significant positive correlation between these variables and the change in RNFL thickness over 27 months (dRNFL). MACQ_27, DASSA_27, and dDASS-A accounted for 35.7% of RFNL variance at 27 months and 21.5% of dRFNL variance.

**Discussion:**

These findings suggest that worse subjective memory complaints and anxiety scores may be associated with one of the most commonly used structural anatomical retinal markers of early disease burden in AD. If so, these results lend support to SMI as a valid risk marker for later cognitive decline.

## Introduction

Alzheimer’s disease (AD) is commonly characterized as a neurodegenerative process gradually progressing from mild cognitive impairment to diffuse cognitive impairment and loss of independence in activities of daily living (Sperling et al., 2011). Within the past two decades, research has revealed an earlier “preclinical stage” of AD, often characterized by elevated cerebral β-Amyloid (Aβ) deposition on positron emission tomography (PET), prior to the development of significant clinical symptoms (Pike et al., 2007). This discovery has propelled a new wave of prevention-based research and clinical drug trials aimed at slowing or halting the onset of AD (Sperling et al., 2011, 2014).

Subjective memory impairment is generally described as self-perceived difficulty with memory or thinking ability and has been associated with depression, anxiety, and prodromal neurodegenerative disease (Abdulrab and Heun, 2008). SMI may be an important feature of preclinical AD that could help predict a future AD diagnosis; however, there are several challenges associated with SMI as a risk marker. First, many instruments have been developed that differentially assess SMI, and the field has yet to agree upon its core features or a universal definition (Molinuevo et al., 2017; Rabin et al., 2017). Additionally, SMI often precedes measurable cognitive changes, making it difficult to characterize objectively for research or for evaluating the efficacy of clinical interventions (Molinuevo et al., 2017; Rabin et al., 2017). SMI also co-occurs with anxiety and depression, which have deleterious effects on memory and cognitive function (Green et al., 2003; Modrego and Ferrandez, 2004) and are uniquely associated with increased likelihood of future AD diagnosis (Yates et al., 2017a, b).

As such, the role of SMI in AD-related cognitive decline is currently under debate. One position is that SMI generally reflects psychiatric overlay such as depression and anxiety and that, as a result, self-reporting is an unreliable metric for predicting cognitive decline (Reisberg and Gauthier, 2008; Reisberg et al., 2008). Conversely, many groups have associated SMI with increased Aβ (Pietrzak et al., 2015), neuroanatomical and metabolic changes (Reisberg and Gauthier, 2008), and decreased cognitive performance (Reisberg and Gauthier, 2008; Koppara et al., 2015).

Comparisons to more well-established AD biomarkers can aid in deciphering what cognitive and subjective symptoms may or may not be associated with AD (Rabin et al., 2015, 2017). Positron emission tomography imaging for neocortical beta-amyloid protein aggregation (Aβ PET) is often used as a reference standard against which the predictive power of other potential biomarkers of disease burden or progression may be compared. Because cerebral amyloid deposition begins many years before symptom onset, it can often identify individuals in the preclinical stage of AD. However, PET imaging is expensive, invasive, and not yet widely available (particularly throughout the developing world). As a result, other lower-cost and less invasive imaging modalities are being explored to identify pathological changes associated with preclinical AD.

Current literature suggests that retinal imaging and visual testing may be promising methods for detecting such changes. Pathological findings may include damage to retinal microvasculature (Heringa et al., 2013) as well as retinal nerve fiber layer (RNFL) thinning (Marziani et al., 2013; Cunha et al., 2017a, b; den Haan et al., 2017). Electroretinogram and curcumin binding studies have revealed increased retinal Aβ and increased latency for the P2 component of visually evoked potentials (VEPs) in AD (Mahajan and Votruba, 2017). A study by Risacher et al. (2013) also identified a potential association between visual contrast sensitivity and AD, MCI, and subjective complaints. More recently, we have reported moderate correlations between the number and surface area of retinal inclusion bodies that we suspect contain fibrillar Aβ, as well as thinning of the RNFL, and PET cerebral Aβ deposition in preclinical AD (Snyder et al., 2016; Santos et al., 2017).

Our previously reported longitudinal cohort study appears to have been the first to examine potential retinal biomarkers of AD in cognitively normal individuals with SMI at increased risk for AD (Snyder et al., 2016; Santos et al., 2017). Here, using the same data repository, we have explored the potential associations between SMI, self-reported mood and anxiety symptoms, and RNFL volumes as a first step toward characterizing the relationship between these variables and how they may relate to the potential for developing AD.

## Materials and Methods

Sixty-three older adults with elevated risk of developing AD (determined by first-degree family history of AD and apolipoprotein E4 (ApoE4) gene expression) were followed for 27 months (Table 1). These criteria (i.e., family history, SMI, and E4 carrier status) were chosen based on the established literature demonstrating their combined association with a 60% likelihood of concurrent cerebral amyloidosis and increased risk for the development of AD (Mielke, 2012). Moreover, these criteria have been used to define preclinical AD cohorts in several prior studies (Kulic and Unschuld, 2016; Snyder et al., 2016; Golzan et al., 2017; Ott et al., 2017). In accordance with the guidelines established by the subjective cognitive decline initiative (SCD-I) working group, the trial was limited to cognitively normal subjects and excluded subjects with mini-mental status exam (MMSE) scores of under 28 (Jessen et al., 2014). Participants were also screened for cognitive impairment using a telephonic cognitive screening test – the minnesota cognitive acuity screen (MCAS) (Tremont et al., 2011). Subjects with a diagnosis of MCI or AD and history of ophthalmologic disease such as age-related macular degeneration (AMD) or cataracts were excluded.

**TABLE 1:** Participant demographic information and baseline, 27-month, and change scores values for self-report variables and RNFL volumes.

**Variable name Variable name**	**Mean**	***SD***	**Min/Max**
Age (years)	63.06	5.42	53, 75
Education (years)	17.21	2.77	12, 24
Sex			
Female	57.14%		
Male	42.86%		
ApoE genotype			
ε2/ε3	12.22%		
ε3/ε3	42.86%		
ε3/ε4	38.78%		
ε4/ε4	6.122%		
ApoE4 gene dose	0.51	0.62	0, 2
MMSE (baseline)	29.00	1.02	27, 30
MMSE (27 months)	29.18	1.34	26, 30
Baseline values			
MAC-Q	26.12	3.27	19, 33
DASS-D	2.73	4.63	0, 22
DASS-A	2.16	3.08	0, 12
DASS-S	5.92	4.70	0, 18
RNFL	0.23	0.02	0.19, 0.28
27-month values			
MAC-Q	21.61	2.64	16, 28
DASS-D	2.65	4.53	0, 26
DASS-A	2.02	3.14	0, 18
DASS-S	4.73	4.18	0, 14
RNFL	0.21	0.02	0.17, 0.30
Change values			
MAC-Q	–4.51	2.93	−10, 3
DASS-D	–0.08	4.51	−17, 17
DASS-A	–0.14	2.87	−8, 11
DASS-S	–1.18	3.92	−10, 9
RNFL	–0.02	0.02	−0.05, 0.04

Subjects completed the 6-item memory assessment clinic questionnaire (MAC-Q) (Crook et al., 1992), 42-item Depression, Anxiety, and Stress Scale (DASS) (Crawford and Henry, 2003), mini-mental status examination (MMSE) (Folstein et al., 1975), and retinal spectral-domain optical coherence tomography study (SD-OCT; SPECTRALIS, Heidelberg Engineering) at baseline and 27 months later. Fourteen subjects were either unable to return for their 27-month end-of-study visits or were unable to complete the retinal imaging component of the study (final sample, *N* = 49, 57% female). Using SD-OCT, RNFL volumes were obtained within the macular region extending 3.45 mm from the foveal center. For each subject, OD and OS values were averaged and mean values were calculated; values for individual eyes were not recorded. Change scores over the 27-month period were obtained for each measure.

Two separate multivariate linear regression models were conducted using STATA (StataCorp College Station, TX, United States), one using RNFL volume at 27 months (RNFL_27), and the other using change in RNFL over 27 months (dRNFL) as dependent variables. Change values were obtained by subtracting baseline RNFL from RNFL at 27 months such that larger dRNFL values signified reduced volume due to RNFL thinning. Independent variables were 27-month MAC-Q scores (MACQ_27), change in MAC-Q scores over 27 months (dMACQ), 27-month self-reported depression, anxiety, and stress scales (DASSD_27, DASSA_27, DASSS_27, respectively), and change in self-reported depression, anxiety, and stress scales over 27 months (dDASSD, dDASSA, and dDASSS, respectively). Change values were obtained for each measure. Co-linearity was assessed using a statistics package in Microsoft Excel. After assessing for co-linearity between age, sex, genetic load, and the previously described independent variables, an additional multivariate regression was conducted to assess the effect of the independent variables with and without age, sex, and other co-variates, respectively, on the dependent variable. Further statistical analysis was conducted on MACQ_27, DASSA_27, and dDASSA due to significant correlation.

This study was approved by and complied with the regulations of the Rhode Island Hospital Institutional Review Board (Lifespan Hospital Systems, Providence, RI, United States). All participants gave written consent in accordance with the Declaration of Helsinki. This study complied with HIPAA regulations.

## Results

An overview of sample results for average baseline, 27-month, and delta values for MAC-Q, DASS, and RNFL testing can be seen below in Table 1. At 27 months, correlations were found between RNFL_27 and MACQ_27 (Adj *R*^2^ = 0.16, *p* < 0.01), DASSA_27 (Adj *R*^2^ = 0.13, *p* < 0.01), and dDASSA (Adj *R*^2^ = 0.25, *p* < 0.01), such that reduced RNFL volume at 27 months was associated with increased memory and anxiety complaints at 27 months. The relationships between 27-month values for RNFL_27 and DASSD_27, DASSS_27, dMACQ, dDASSD, and dDASSS were not significant.

There was also a significant positive correlation between dRFNL and MACQ_27 (Adj *R*^2^ = 0.06, *p* = 0.05), DASSA_27 (Adj *R*^2^ = 0.10, *p* = 0.02), and dDASSA (Adj *R*^2^ = 0.11, *p* = 0.01) such that RNFL volume loss over 27 months was associated with increased memory and anxiety complaints at 27 months. There were no significant correlations between dRNFL and DASSD_27 (*p* = 0.75), DASSS_27 (*p* = 0.52), dMACQ (*p* = 0.28), dDASSD (*p* = 0.54), or dDASSS (*p* = 0.78).

**FIGURE 1:**
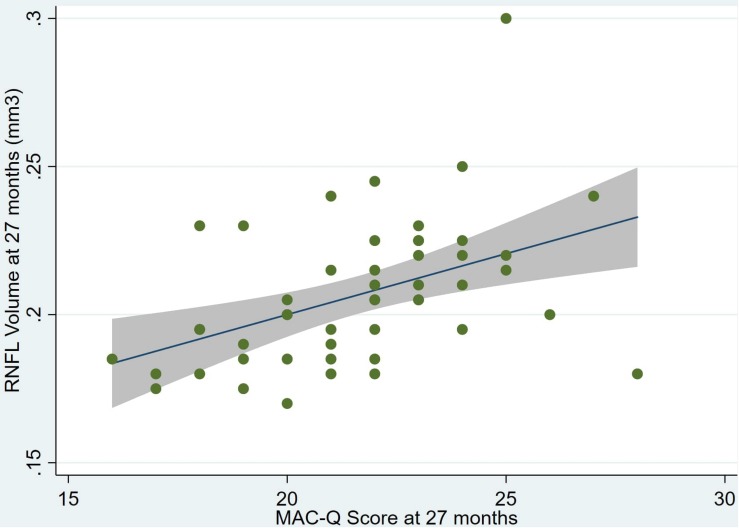
Individual regression models for MACQ_27 using baseline RNFL volumes (RNFL) as the dependent variable.

**FIGURE 2:**
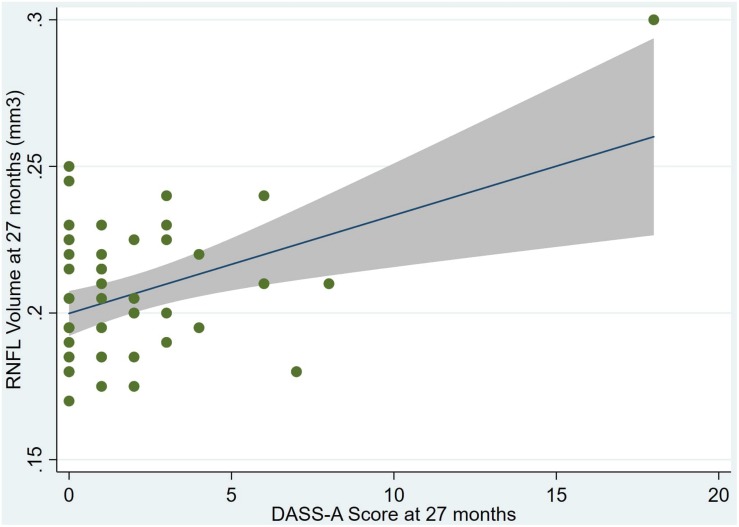
Individual regression models for DASSA_27 using baseline RNFL volumes (RNFL) as the dependent variable.

**FIGURE 3:**
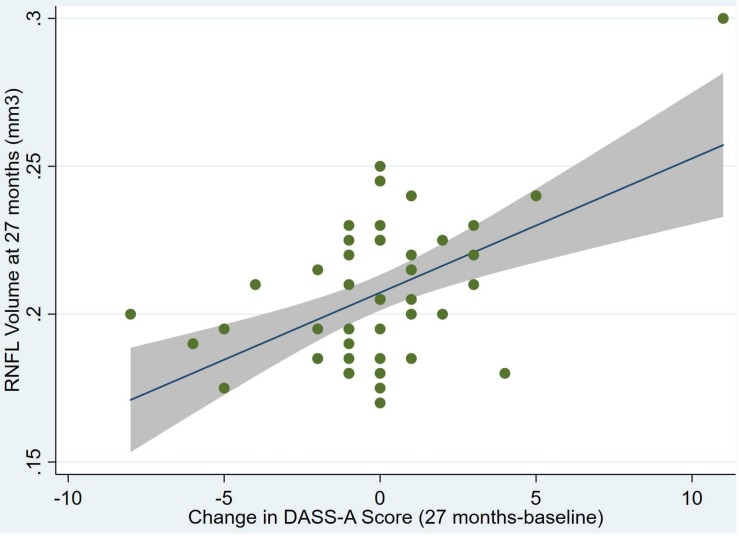
Individual regression models for dDASSA using baseline RNFL volumes (RNFL) as the dependent variable.

**FIGURE 4:**
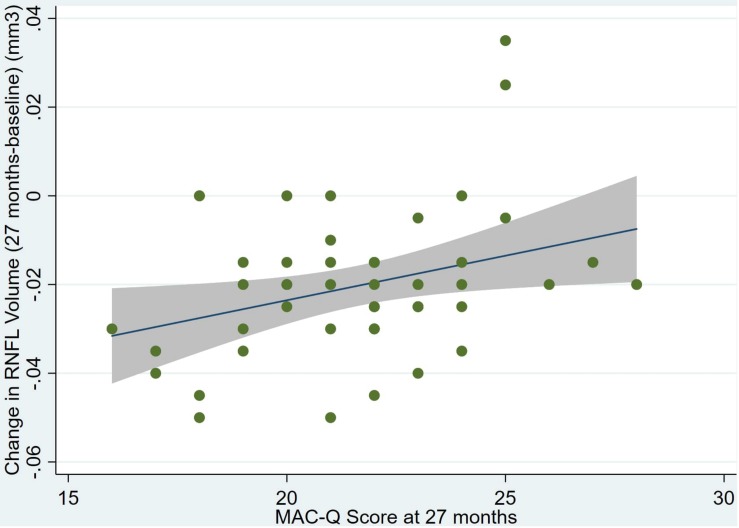
Individual regression models for MACQ_27 using change in RNFL volume (dRNFL) as the dependent variable.

**FIGURE 5:**
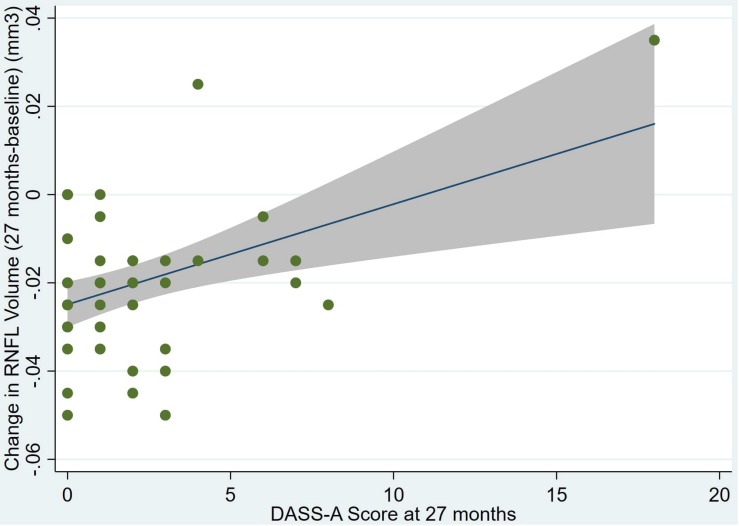
Individual regression models for DASSA_27 using change in RNFL volume (dRNFL) as the dependent variable.

**FIGURE 6:**
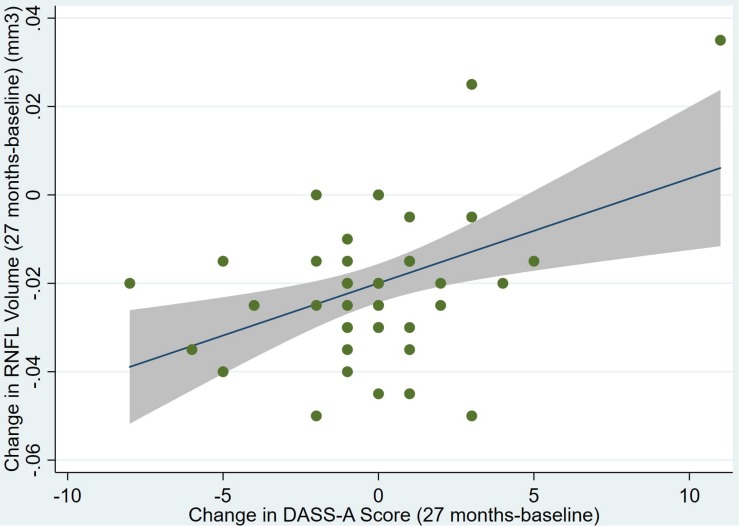
Individual regression models for dDASSA using change in RNFL volume (dRNFL) as the dependent variable.

Individual regression models can be seen in Figures 1–6 below. These individual regression models identify possible outliers on DASSA_27 and dDASSA testing. When removing one outlier whose DASSA_27 score was 18 (compared to average DASSA_27 2.02), the correlation between DASSA_27 and RNFL and between DASSA_27 and dRNFL is not significant (*p* = 0.96 and *p* = 0.93, respectively). The correlation between dDASSA and RNFL and between dDASSA and dRNFL remains significant (*p* = 0.05, *p* = 0.03, respectively) after removal of one significant outlier. Importantly, there were no statistically significant correlations between either DASSA_27 or MACQ_27 with MMSE scores at 27 months or with change in MMSE scores over 27 months (*p* > 0.05). Assessment of co-linearity demonstrated mild to moderate correlations between DASS-S, DASS-D, DASS-A, and MAC-Q scores ranging from 0.07 to 0.46 suggesting that these variables are separate but related constructs; of these, only correlations between DASS-D and DASS-S, DASS-A and DASS-S, and DASS-A and MAC-Q were significant (*p* = 0.01, *p* = 0.01, p < 0.01, respectively). All other correlations between anxiety, depression, stress, and cognitive scores were not significant (*p* > 0.05).

Further multivariate regression models were conducted on statistically relevant variables MACQ_27, DASSA_27, and dDASSA. Statistical results from the multivariate models can be seen in Tables 2, 3. Together, MACQ_27, DASSA_27, and dDASSA accounted for approximately 35.7% of RNFL_27 variance (Adj. *R*^2^ = 0.36; *p* < 0.01) and approximately 21.5% of dRFNL variance (Adj. *R*^2^ = 0.22; *p* < 0.01). For RFNL_27, the effects of MACQ_27 and dDASSA were significant (*p* = 0.02, *p* < 0.01) while DASSA_27 was not (*p* = 0.35). For dRFNL, the effect of MACQ_27 was significant (*p* = 0.02), while DASSA_27 and dDASS-A were not (*p* = 0.11 and *p* = 0.25, respectively). When accounting for age, sex, or APOE4 status, there was no significant change in variance of RNFL_27 or dRNFL.

**TABLE 2:** Statistical results from multivariate model with RNFL_27 as a dependent variable.

	**Coefficient**	**SE**	***T*-Score**	***P*-Value**	**95%CI:**	**95%CI:**
					**Low**	**High**
MACQ_27	0.15	0.02	5.93	4E−07	0.10	0.20
DASSA_27	1.02E−3	1.08E−3	0.95	0.35	−1.14E−3	3.19E−3
dDASSA	2.70E−3	1.16E−3	2.32	0.02	0.36E−3	5.04E−3

**TABLE 3:** Statistical results from multivariate model with dRNFL as a dependent variable.

	**Coefficient**	**SE**	***T*-Score**	***P*-Value**	**95%CI:**	**95%CI:**
					**Low**	**High**
MACQ_27	−0.04	0.02	–2.41	0.02	−0.08	−0.01
DASSA_27	1.33E−3	0.80E−3	1.65	0.11	−0.29E−3	2.94E−3
dDASSA	1.00E−3	0.87E−3	1.16	0.25	−0.74E−3	2.75E−3

## Discussion

Recent studies suggest that RNFL thinning is potentially associated with increased cerebral amyloid deposition in the preclinical stage of AD and may have potential as a surrogate biomarker for AD, if sufficiently validated through further research. The goal of this study was to assess whether subjective cognitive and psychiatric complaints that have also been associated with the early stages AD are correlated with lower RNFL volumes due to thinning of the RNFL over time.

In this study, we followed 49 cognitively normal older adults with increased AD risk for 27 months using SD-OCT, MMSE, MAC-Q, and DASS. We found that both worse reported anxiety at 27 months, as well as increases in self-reported symptoms of anxiety over 27 months, were associated with reduced RNFL volume at 27 months. Similarly, worse SMI scores at 27 months were associated with reduced RNFL volume and increased RNFL thinning. These data suggest that SMI and self-reported anxiety are associated with RNFL volume and RNFL thinning, two retinal biomarkers previously shown to correlate with cerebral Aβ deposition.

This is an initial report with a relatively small sample size. Future work may indicate whether elevated DASS-A scores reflect anxiety as a direct result of SMI, an association that is challenging to evaluate with self-report questionnaires. Notably, baseline values for RNFL and MAC-Q, DASS-D, DASS-A, and DASS-S demonstrated positive correlations with each other, but were not statistically significant, and hence effect magnification over time may have contributed to our findings, to a degree.

Interestingly, unlike anxiety, baseline, delta, and 27-month scores for self-reported stress and depression were not associated with RNFL volume or changes in RNFL volume. This finding may be consistent with prior research suggesting that anxiety is uniquely predictive of cognitive decline and AD (Teri et al., 1999; Sinoff and Werner, 2003; Balash et al., 2013). For example, Pietrzak et al. (2015) found that high anxiety, Aβ + older adults demonstrated a more precipitous cognitive decline compared to those with low anxiety or depression alone. These findings suggest that self-reported anxiety may play a significant role in preclinical AD and future cognitive decline, even when compared to measures of depression or stress.

A common concern noted, with respect to reliance on SMI as a risk marker for preclinical AD, is the confounding factor of true cognitive decline (CD). In order to control for this in the current study, we required an MMSE score of >28. We did not find any significant change in MMSE over the 27-month period, and we likewise did not find any correlation between MMSE and MAC-Q/DASS. This supports the hypothesis that SMI may play a role in preclinical AD independent from objective cognitive impairment.

However, there are several limitations to this study. First, our analysis was limited to 49 subjects followed over the span of 27 months. Replication in a larger cohort followed over a longer span of time will be necessary to establish the strength and generalizability of these findings. The study cohort was limited to cognitively normal subjects characterized as high-risk for developing AD based on first-degree family history, ApoE4 carrier status, and SMI, but the presence of biomarker-defined (i.e., Aβ PET) preclinical AD could not be confirmed, which limits the interpretation of our findings to a degree. Future studies associating SMI and retinal biomarkers to established AD biomarkers (e.g., tau and amyloid) in CSF or PET imaging data are therefore an important next step for this line or research. Moreover, our high-AD risk sample characterizes only one subset of individuals who may develop AD, and our findings may not extend to the broader older adult population characterized by more variable levels of AD risk. Future studies would ideally include comparison groups of ApoE4 positive subjects with clinical disease as well as cognitively normal subjects without risk factors for AD in order to clarify the generalizability of these findings. Although we did not observe any significant effects of age, sex, or APOE status in our analyses, these variables could still emerge as important moderators with a larger sample. Finally, recent studies have suggested that retinal thinning in AD may be more pronounced in the superior hemiretina compared to the perimacular retina; therefore, further research may benefit from imaging larger retinal areas (den Haan et al., 2017). Overall, additional research is needed to provide further validation as RNFL as an AD biomarker.

Despite these limitations, we consider this current report to be “path finding” in suggesting a novel relationship between subjective memory impairment (SMI), anxiety, and RNFL changes in older adults at high risk for AD. As the field of research in preclinical AD continues to expand, future studies may further validate these findings. Though there are inherent challenges with the use of SMI in both research and clinical practice, we suggest that SMI may potentially be a reasonable adjunct to objective biomarkers of preclinical AD, especially when examined in concordance with literature-established guidelines.

## Conclusion

The role of SMI in preclinical AD has been the subject of continuing debate in the literature. In this study we found a significant correlation between SMI (as measured by the memory assessment questionnaire and depression, anxiety, and stress scales) and RNFL volume– metrics that have previously been implicated in preclinical AD (Lu et al., 2010; Snyder et al., 2016; Santos et al., 2017; Ko et al., 2018; Sanchez et al., 2018). This supports the potential use of SMI as a risk marker for preclinical disease and its incorporation into the developing array of preclinical AD assessment tools. Further validation of SMI with other preclinical assessment tools such as PET imaging, macular pigment optical density (MPOD), and retinal vasculature imaging (e.g., measurement of vascular bed complexity and tortuosity) is needed. Despite these challenges, our findings suggest that SMI should be considered as a reasonable risk marker, to augment other more objective methods, of screening for preclinical AD.

## Data Availability Statement

The datasets for this manuscript are not publicly available because they include protected health information. The datasets generated for this study are available on request to corresponding author.

## Ethics Statement

This study was approved by and complied with the regulations of the Rhode Island Hospital Institutional Review Board (Lifespan Hospital Systems, Providence, RI, United States). All participants gave written consent in accordance with the Declaration of Helsinki. This study complied with HIPAA regulations.

## Author Contributions

DC and LT contributed to the data gathering and analysis, and manuscript preparation and revision. PS contributed to the study design, data gathering and analysis, and final manuscript preparation.

## Conflict of Interest

The authors declare that the research was conducted in the absence of any commercial or financial relationships that could be construed as a potential conflict of interest.
